# Treatment of pathologic spinal fractures with combined radiofrequency ablation and balloon kyphoplasty

**DOI:** 10.1186/1477-7819-7-90

**Published:** 2009-11-16

**Authors:** Pavlos Katonis, Dritan Pasku, Kalliopi Alpantaki, Artan Bano, George Tzanakakis, Apostolos Karantanas

**Affiliations:** 1 Department of Orthopaedic and Traumatology, University Hospital of Heraklion, Crete, Greece; 2 Department of Histology, Medical School, University of Crete, Heraklion, Greece; 3 Department of Radiology, University Hospital of Heraklion, Crete, Greece

## Abstract

**Background:**

In oncologic patients with metastatic spinal disease, the ideal treatment should be well tolerated, relieve the pain, and preserve or restore the neurological function.

The combination of fluoroscopic guided radiofrequency ablation (RFA) and kyphoplasty may fulfill these criteria.

**Methods:**

We describe three pathological vertebral fractures treated with a combination of fluoroscopic guided RFA and kyphoplasty in one session: a 62-year-old man suffering from a painful L4 pathological fracture due to a plasmocytoma, a 68-year-old man with a T12 pathological fracture from metastatic hepatocellular carcinoma, and a 71-year-old man with a Th12 and L1 pathological fracture from multiple myeloma.

**Results:**

The choice of patients was carried out according to the classification of Tomita. Visual analog score (VAS) and Oswestry disability index (ODI) were used for the evaluation of the functional outcomes. The treatment was successful in all patients and no complications were reported. The mean follow-up was 6 months. Marked pain relief and functional restoration was observed.

**Conclusion:**

In our experience the treatment of pathologic spinal fractures with combined radiofrequency ablation and balloon kyphoplasty is safe and effective for immediate pain relief in painful spinal lesions in neurologically intact patients.

## Background

The spine is the most frequent site of bone metastases. Spinal involvement may occur in up to 40% of patients with cancer and approximately 70% of patients with cancer have evidence of metastatic disease at the time of their deaths [1]. As many as 75% of vertebral metastases occur in patients with carcinoma of breast, kidney, lung, prostate, thyroid, and multiple myeloma [2,3]. The management of metastatic spinal disease aims at pain control, maintenance or restoration of neurologic function and stability [4]. Standard treatments include radiation therapy, chemotherapy and surgery. Minimally invasive therapeutic options, including kyphoplasty and radiofrequency ablation have recently been introduced for the treatment of painful spinal metastases. Percutaneous kyphoplasty is a fluoroscopic guided procedure that consists of percutaneous insertion of a balloon, which creates a cavity followed by intravertebral installation of PMMA. It can be used in both benign spinal disorders such as osteoporotic or traumatic fractures and malignant lesions such as metastases [5,6]. It provides significant pain relief as well as reinforcement and stabilization of the bone [7]. Radiofrequency ablation (RFA) is a relatively new method for the treatment of painful bone metastases. It was initially introduced for the treatment of osteoid osteoma [8] and then became an important alternative and safe method in the treatment of metastatic liver, renal and lung tumors [9-11].

The aim of this article is to present the feasibility of performing the combined RFA and balloon kyphoplasty (BKP) in 3 patients with painful spinal metastatic disease in one session.

## Materials and methods

Three patients with painful metastatic foci in the spine were included in the study which was in accordance with the guidelines of the Helsinki Declaration and informed consent was obtained in each case.

### Surgical Technique

**Figure 1:**
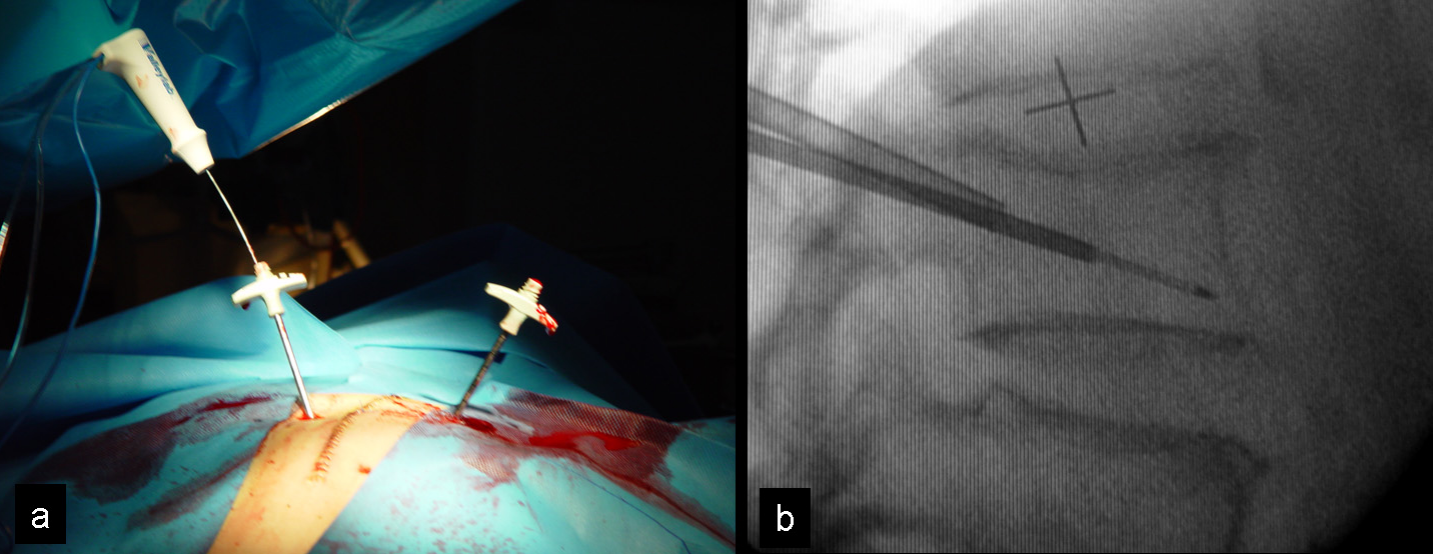
**The two needle approach as shown in the theatre (a) and fluoroscopy (b)**.

In all cases, the treatment was performed by orthopaedic surgeons. All patients had normal coagulation blood test platelets levels before treatment. Under general anesthesia, the patient was placed in a prone position and the skin was prepared with povidine iodine (10%) solution. Two small incisions were made to access the vertebral pedicles. Biplanar fluoroscopy, using two C-arms devices, was preferred for better visualization of the needle placement and the PMMA (Kyphon, Medtronic, Minneapolis, MN, USA) cement flow. Under fluoroscopy guidance, two biopsy needles were introduced transpedicularly into the involved vertebra (Fig 1a). A singular internally cooled, 17 gauge electrode with 1 cm exposed tip and 15 cm length (Valleylab, Boulder, CO, USA) was inserted through a cannula into the vertebral body from one side (Fig 1b). Tissue specimens for histological examination were obtained in all cases before RFA (in the third case we obtained tissue for histological examination before and after RFA). The net ablation time was 8 min at an energy level of 60 W with a target temperature between 80°-90°C. In all cases the surgical operations were performed with a 480 kHz generator model CC-1 (Radionics, Burlington, MA, USA).

**Figure 2:**
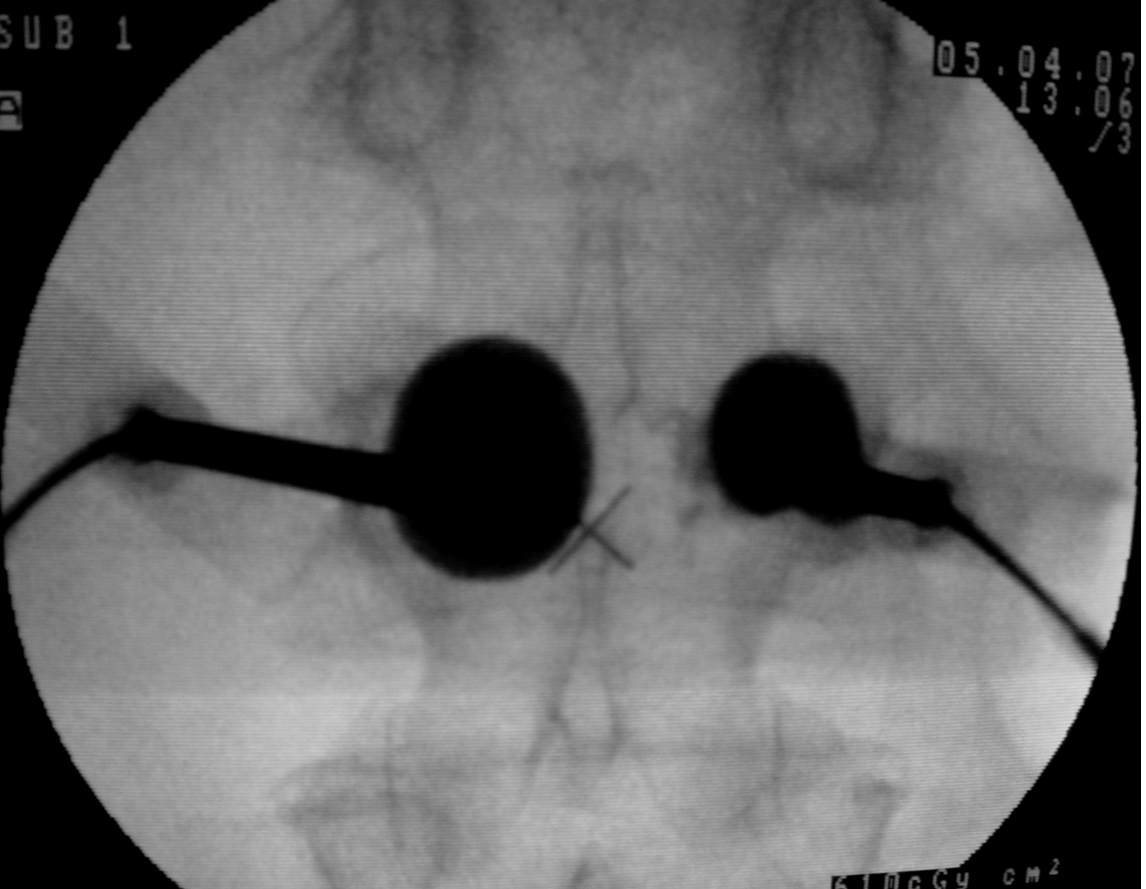
**Under fluoroscopic control, the inflation of the balloons is shown**.

After the ablation step, the radiofrequency probes were removed and the kyphoplasty balloons (Kyphon, Medtronic, Minneapolis, MN, USA) were inserted and inflated (Fig 2). Partial reduction of the collapsed vertebral body was possible in all cases. At the same time the PMMA was prepared. PMMA was filled in the special cannula and then injected about 9-10 min after the preparation into the osseous cavity created by the balloons and controlled by fluoroscopy.

### Case 1

**Figure 3:**
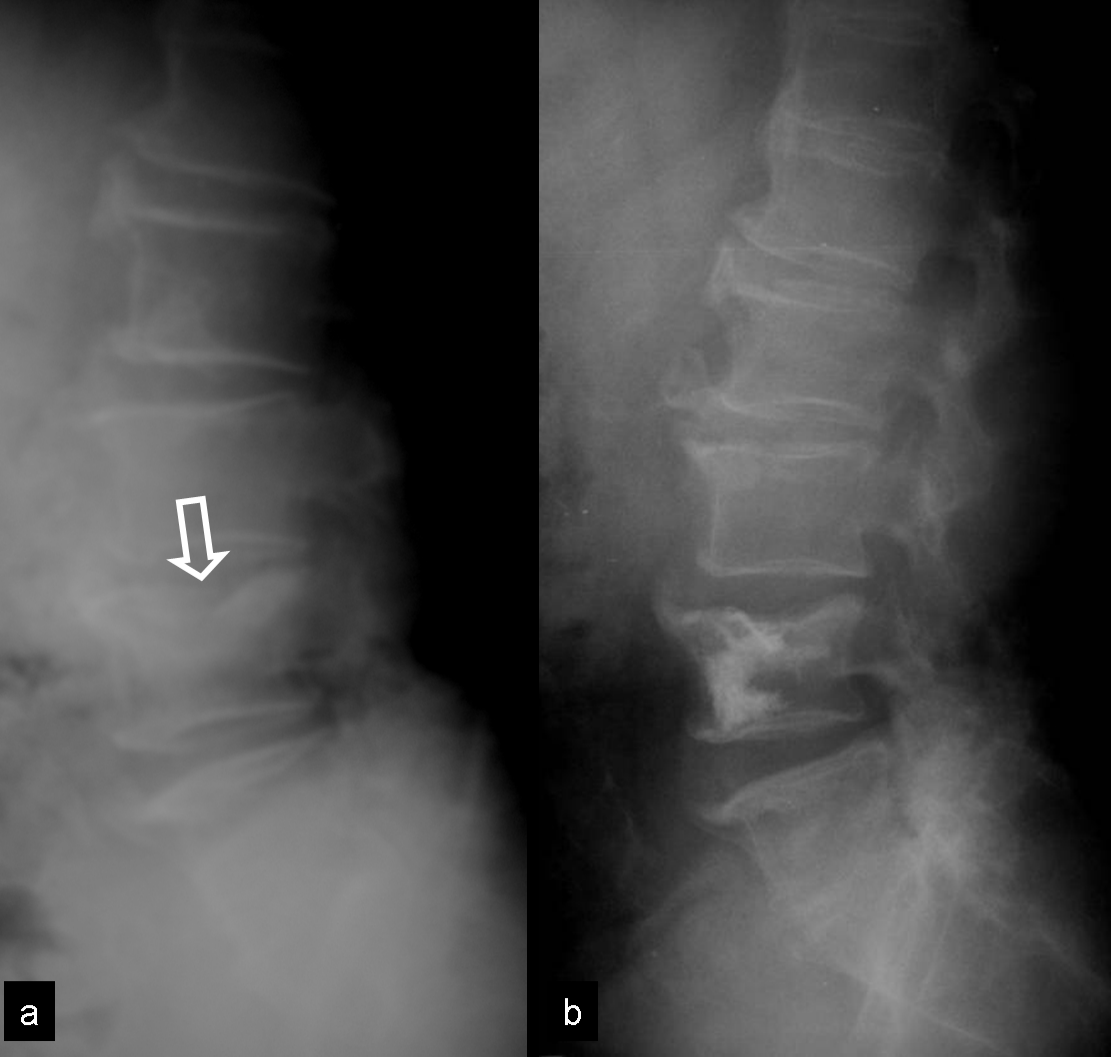
**A 62-year-old man with persistent low back pain**. a) The lateral x ray of the lumbar spine shows the collapse of the superior endplate of the L4 vertebral body (arrow). b) The postoperative x ray shows the results of the combined ballon kyphoplasty and radiofrequency ablation, with height restoration.

A 62-year old man with severe back pain was admitted to tertiary care in a specialized spinal unit at the University Hospital. He complained of serious low back pain during the last 6 months without any improvement following conservative management. There was no history of trauma and the patient was neurologically intact. The serologic examination revealed an increased ESR (87 mmHg). The plain x rays (Fig 3a) and the MR imaging of the lumbar spine showed a pathologic fracture of the L4 vertebral body, mainly involving the superior epiphyseal plate, without extension into the spinal canal.

The preoperatively referred VAS and the Oswestry disability index (ODI) were 6 and 66% respectively. The quantity of the preoperative analgesic therapy was registered. According to the classification of Tomita et al. [12], the patient had a score of 3, justifying a long term therapy. In consensus with the haematologists, a combined RFA and BKP of L4 vertebra was performed (Fig 3b) (see the surgical technique). The radiofrequency electrode was introduced two times in order to reach a larger necrotic area. The volume of the injected PMMA cement was 4 ml. An increase in 15% of the mid vertebral body height was observed. The histological examination confirmed the diagnosis of plasmacytoma. No complications related to procedure were observed. Postoperatively, the VAS and and Oswestry scores were 1% and 28% respectively and the patient did not need any additional analgesic therapy. In the 7-months follow-up, the patient was free of pain, the primary disease being stable with VAS and ODI being 2% and 40% respectively.

### Case 2

A 64-year-old man with a known liver cancer diagnosed 6 months ago, was presented to our clinic with severe pain in the lower thoracic region, deteriorating at night without any neurological deficit. He was neurologically intact. His medical history revealed a stable coronary heart disease. Histologically, the tumor was characterized as a hepatocellular carcinoma with moderate growth complicated with a treatable lung metastasis during the investigation after hospitalization. According to the classification of Tomita et al. [12], the patient with 6 points in the scoring system, was an eligible candidate for a palliative surgery, aiming at short-term local control. Preoperatively his VAS score was 8 and his Oswestry low back pain disability questionnaire 84%.

**Figure 4:**
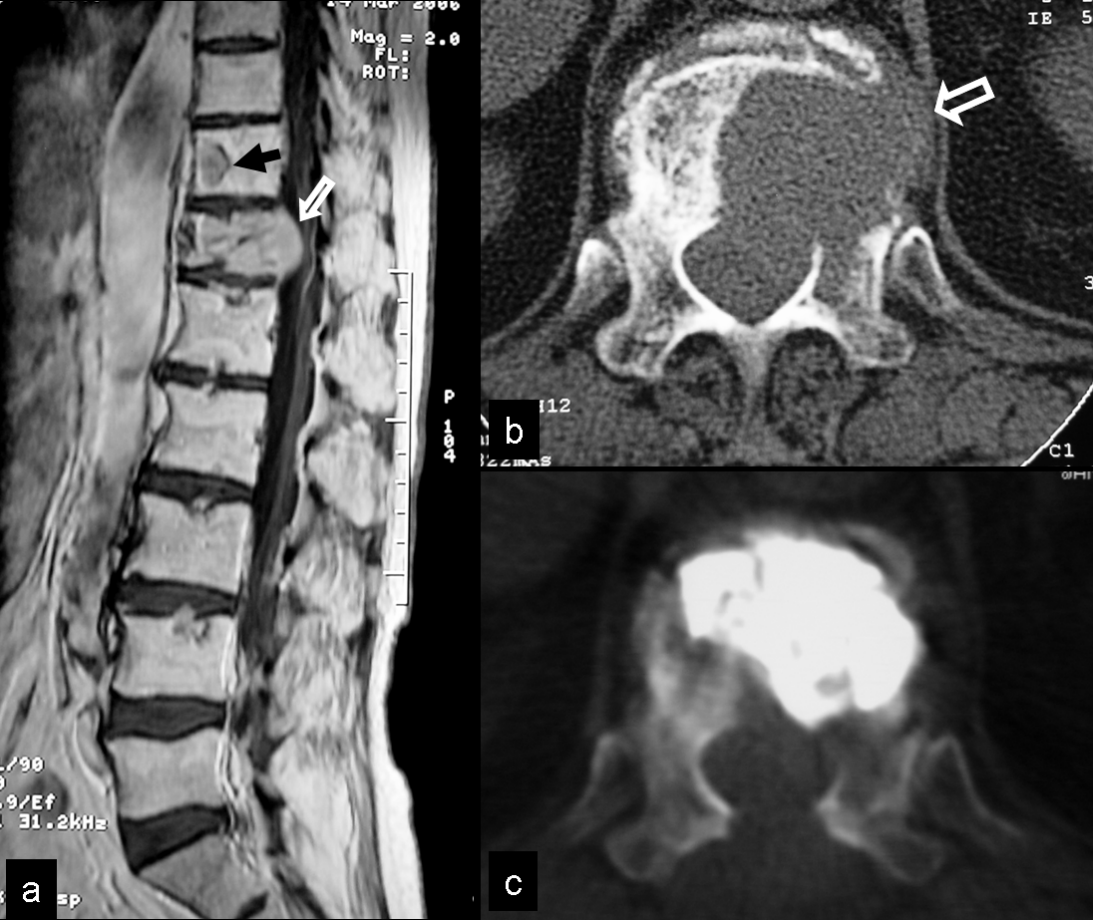
**Malignant fracture of the T12 vertebral body, secondary to hepatocellular carcinoma**. α) The sagittal contrast enhanced T1-w TSE MR image shows the malignant fracture with posterior displacement (arrow). An additional secondary deposit is shown in the T11 vertebral body (black arrow). The axial CT images show the preoperative osteolysis (arrow in b) and the postoperative result (c).

The plain x-rays and the MR image show the malignant fracture at T12 level with moderate posterior bulging into spinal canal (Fig 4a,b). An additional secondary deposit is shown in the T11 vertebral body. RFA and BKP of the lesion was performed. The radiofrequency electrode was introduced two times into the body and one time in the left isthmus area of the Th12 vertebrae. The volume of the injected PMMA cement during BKP was 4 ml (Fig 4c). The placement of the PMMA is performed very carefully after 10 min of preparation and under continued fluoroscopic image in order to avoid the symptomatic extravasation. Increase of 8% and 11% were measured respectively for the anterior and middle vertebral wall. RFA only was performed in the T11 vertebrae and the surgical treatment was completed with the laminectomy of the T12 vertebra in order to avoid the challenging anterior approach and reconstruction in an already aggravated patient.

Immediately post-operatively, the VAS score was 4 and the need for painkillers was drastically reduced. No complications related to the procedure were observed. The VAS and the ODI at the 3-months follow-up were 2 and 52% respectively. The patient died 6 months after treatment from complications related to portal hypertension, being neurogically intact.

### Case 3

**Figure 5:**
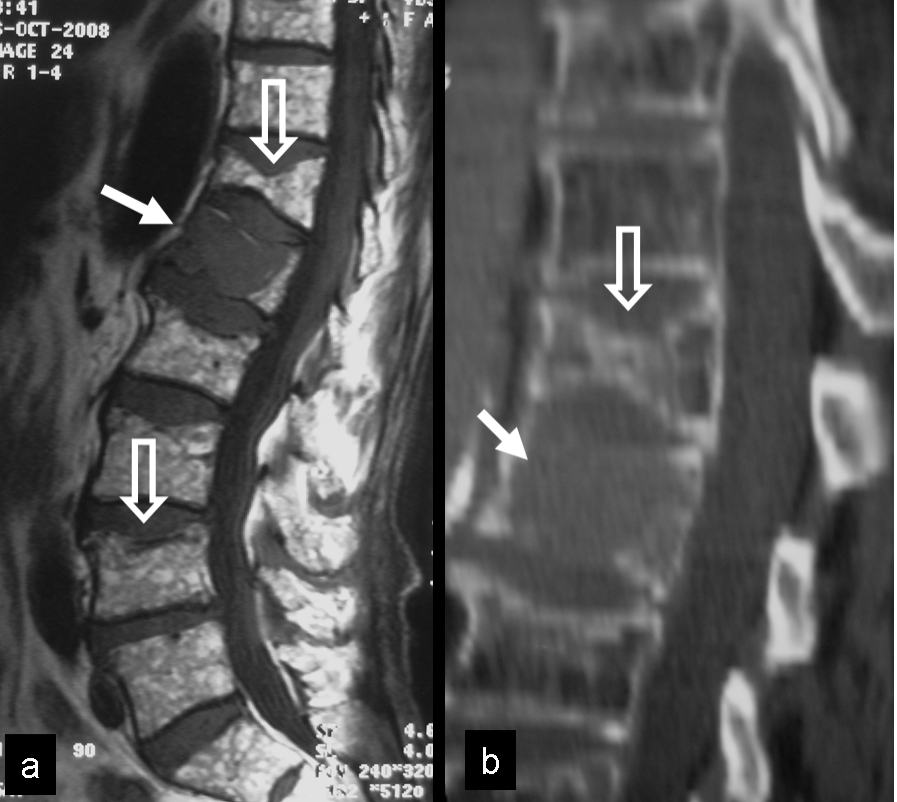
**Diffuse thoracolumbar pain in a 71-year-old patient**. The T1-w TSE MR image (a) and the sagittal reconstruction of the corresponding CT, showed diffuse infiltration of the L1 vertebral body (arrows). In addition, old osteoporotic fractures were noticed in the T12 and L4 vertebrae (open arrows). The bone marrow signal intensity on the MR image is inhomogenous.

A 71-year-old man was admitted with invaliding, diffuse pain in the thoraco-lumbar region. The patient had reported weight loss and appetite disorders during the last 3 months. A diagnosis of diabetes mellitus type II had been established 8 years ago. The serologic examination showed hypercalcaemia (10.3 mg/dl) and increased ESR (101 mmHg). MR imaging and CT of the lumbar spine showed diffuse infiltration of the L1 vertebral body (Fig 5a,b). In addition, old osteoporotic fractures were noticed in the T12 and L4 vertebrae. The bone marrow signal intensity on T1-w images was inhomogeneous, suggesting either a chronic anaemia with red marrow reconversion or diffuse metastatic disease. Preoperatively, the VAS and the ODI were 9 and 78% respectively.

**Figure 6:**
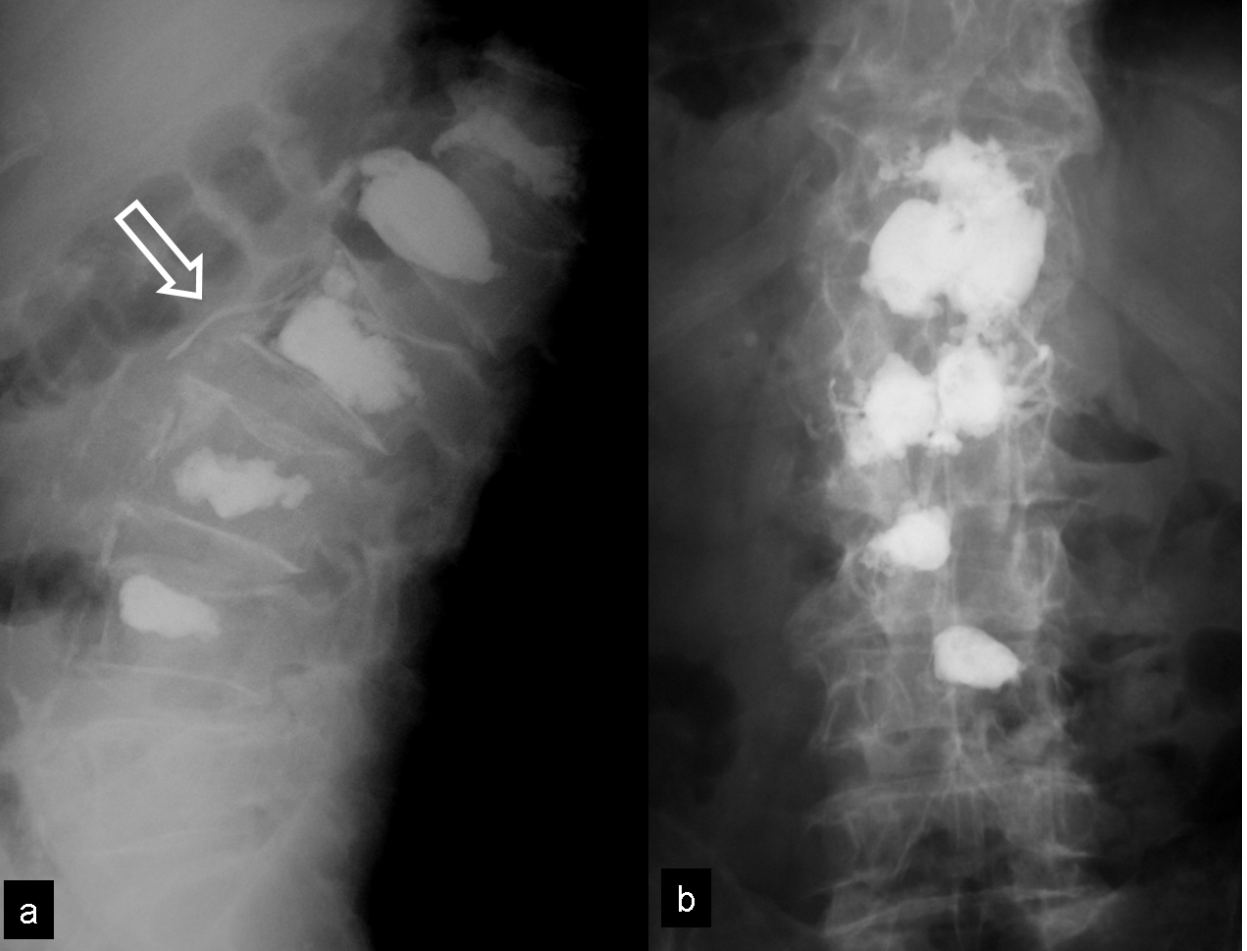
**The lateral and AP x rays of the lower spine show the postoperative results (case 3)**. An intravenous cement extravasation from L2 vertebra is shown (arrow).

**Figure 7:**
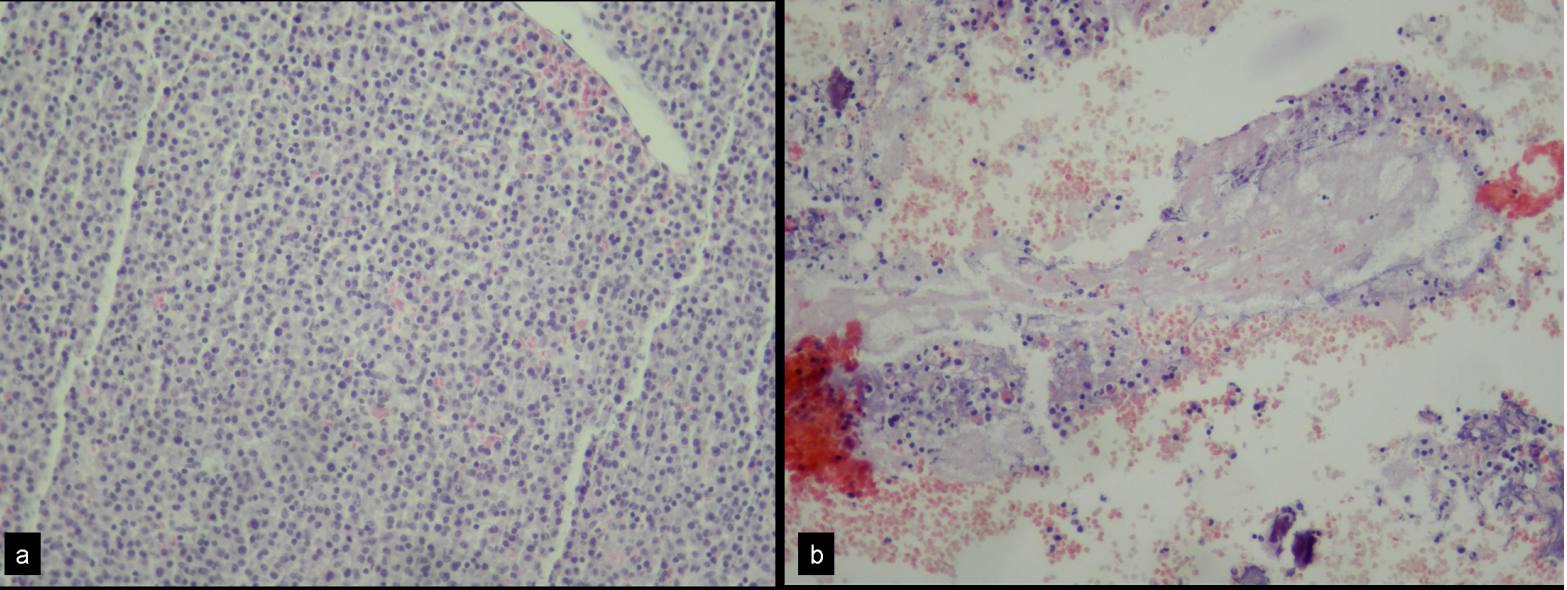
**a) The histopathological examination of the third patient before the radiofrequency shows a large quantity of monoclonal myelomatous cells (×200, H&E)**. b) The corresponding examination after the ablation shows a significant reduction of the myelomatous cells with diffuse areas of necrosis (×200, H&E).

According to the classification of Tomita et al. [12], the patient had a score of 4. RFA combined with BKP were planned for the T12 and L1 vertebrae and BKP alone in L2, L3 and L4 (Fig 6a, b). The radiofrequency electrode was introduced two times in order to have a larger necrosis area in both T12 and L1 vertebral bodies. In this patient, material for histopathological examination was taken before (Fig 7a) and after radiofrequency ablation. Radical depletion of the number of the monoclonal myelomatosus cells after the RFA with diffuse necrosis was shown (Fig 7b). The amount of the injected cement was 2,5 ml for T12 and L3 vertebra, 5 ml for L1, 4 ml for L2 and 2 ml for L4 vertebra.

Increases of 6% and 12% were measured respectively for anterior and middle wall of T12 vertebra and 10% and 18% were measured respectively for anterior and middle wall of L1 vertebra. 13° restoration of the kyphotic angle was observed.

An asymptomatic intravenous cement extravasation from L2 vertebra (fig 6a) was observed. The patient presented a low-grade fever (<38°C) for two days post-operatively without any additional symptoms and signs of infection.

The directly post-operatively VAS score was 4 whereas in the 6-month follow-up the VAS and Oswestry scores were 2% and 36% respectively, without any consumption of analgesics.

## Discussion

Spinal complications of osseous metastatic disease may have detrimental effects on the quality of life of cancer patients. The treatment of patients with symptomatic metastatic spinal disease is performed in order to relieve the pain and to preserve or restore the neurological function. Cure is not usually a realistic expectation as the life expectancy is often relatively short with medial survival ranging from 4 to 15 months in different series [12-14]. The decision to provide surgical treatment is complex and debatable. Many authors believe that patients with a good prognosis benefit from radical surgical treatment [4]. For preoperative evaluation we have used the classification of Tomita et al. namely the grade of malignancy and the presence of bone metastases and/or visceral metastases [12]. Many authors find it simple to use in the management of spinal metastases [15]. The anterior approach remains the "gold standard", but a posterior approach might be obligatory for certain patients [12,15].

In this group of patients, an aggressive surgical attitude may be associated with substantial complications further classified as surgical (e.g., wound infections, haematomas, cerebrospinal fluid fistulas), instrumentation failures (broken, misplaced, migrated), medical (cardiovascular, pulmonary, nutritional), and neurologic (i.e., neurologic deterioration) [16]. Currently, new evolving surgical techniques have enabled spinal surgeons to provide safe and improved management to the overall disease. Among the latter, RFA and vertebral body augmentation (vertebroplasty and kyphoplasty) have been employed for the treatment of spinal metastatic tumors with good results [17,18].

The term "ablation" refers to the local destruction of the tumor by the means of application of either chemical agents (ethanol, acetic acid), or local deposition of some form of energy (radiofrequency, cryoablation, microwave, laser, and ultrasound). RFA is described in the literature as a safe and effective therapy to enhance the local control of malignant disease [11]. In tumoral spine disease, indications for BKP include compression fractures from multiple myeloma and metastatic disease, particularly when conservative treatment has failed. Contraindications for BKP may include: (1) uncorrectable coagulopathy (2) pain unrelated to vertebral collapse 3) osteoblastic metastasis 4) severe iodine allergy 5) technically not feasible tumoral vertebral lesion [19,20].

Kyphoplasty offers theoretical advantages over vertebroplasty regarding the restoration of the collapsed vertebral height and the reduction of the possibility of symptomatic extravasation [19-21]. Additionally, in extensive osteolytic lesion, the eggshell kyphoplasty technique minimizes the possibility of symptomatic leakage into the spinal cord and the neuronal foramina [21,22]. However nowadays, there is no definitive conclusion about which technique is preferable because of the lack of sufficiently randomly controlled trials.

The aim of the combination of the RFA with kyphoplasty is threefold; firstly, to achieve a reduction of pain. The radiofrequency has a destructive effect over the sensory nerve fibers in the periosteum, which could result in an inhibition of the pain transmission [23]. In addition, PMMA which has a mechanically stabilizing effect plays an important role in pain score improvement.

Secondly, to reinforce the antitumoral effect. RFA has a strong catabolic effect on tumor cells that are producing nerve-stimulating cytokines such as tumor necrosis factor-alpha (TNF-α), interleukins (IL-1 and IL-6), resulting in inhibition of osteoclast activity. PMMA has a direct toxic effect on the neoplasic cells due to its chemical and thermal properties [20,21,24].

Thirdly, the combination of the vertebral body augmentation matched with the antitumoral effect, might prevent a potential neurologic impairment secondary to a further collapse of the vertebra [8,21].

In addition, RFA, which causes vascular necrosis and thrombosis in highly vascularized metastatic tumors, might reduce the risk of symptomatic vascular leakage during the cement augmentation.

To the best of our knowledge, the combination of RFA with BKP has not been described previously for the treatment of metastatic spinal fractures. In this limited number of patients, a significant reduction in pain scores and improvement in quality of life following the treatment with RFA and kyphoplasty for painful spinal metastases was noted. An associated significant reduction in opioid requirements was also noted at 3 and 6 months postoperatively.

Other studies have shown an important improvement in the Oswestry Disability Index (ODI) and VAS score in patients treated with kyphoplasty for osteolytic compression fractures resulting from multiple myeloma [25,26].

No leakage of cement was seen intraoperatively or in the post-operatively radiological examinations. Kyphoplasty has a lower rate of cement leakage than vertebroplasty making the cement augmentation safer [20,27,28]. A recently updated meta-analysis reported a range of leakage after BKP (balloon kyphoplasty) up to 21.8% however the range of leakage after vertebroplasty series was estimated to be from 2% to 67% [29].

Pflugmacher et al. report 12% of cement leakage after 99 BKP procedures in patients with spinal metastatic disease [30]. Importantly, the incidence of intravenous leaks and systemic embolization are similar for both procedures [28,29,31]. Naturally, the safety of the procedure has a crucial importance in these patients with an already aggravated medical situation. We applied the egg-shell technique in order to minimize the cement leakage through the involved anterior or lateral wall and in case of serious defect of the posterior wall, the placement of the PMMA is performed very carefully after 10 min of preparation and under continued fluoroscopic image in order to avoid the symptomatic extravasations.

In our study BKP was associated with a partial restoration of the vertebral height and kyphotic angle post-operatively and in the 3-month period of follow-up. However, height restoration and kyphotic correction are not the first priority in this group of patients. Recently, Liu et al. have demonstrated that BK is more effective in vertebral body augmentation than vertebroplasty [32]. In an another study the improved radiographic outcomes of vertebral height restoration were observed to return to the pre-operative levels 12 months after the operation for metastatic spine disease [30].

After RFA, a considerable reduction of pain and improvement in the quality of life, as well as a decrease in the use of analgesic medications, is reported [24,32,33]. But, we were unable to find any histopathologic documentation of the antitumoral action of the RF. A rate of 10% of complications occurred during the RFA for liver lesions was reported [11]. In spinal ablation the rate of complication is unknown. No complications including neurological structure damage, skin burns, infection or hemorrhage, related to RFA [11], were observed intra-operatively or early post-operatively in patients presented herein. We have not observed postoperatively the so-called post-ablation syndrome consisting of low-grade fever, myalgias, and malaise for up to 1 week after the procedure.

In the spine, RFA may be contraindicated due to the close relationship with the neural structures [24,33,34]. It has been suggested, and we adopted this approach, that the placement of the probe in the center of the vertebral body is a safe way to minimize such complications at the shortest distance from the spinal cord 1 cm [33,34]. In order to enlarge the coagulation area, we placed the electrode twice into each involved vertebra. The histopathological examination in the third case demonstrated that RFA is efficient in inducing tumoral cells necrosis. The consequent toxic effect of PMMA during BKP acts synergically in tumoral cells necrosis.

To minimize the cytotoxic effects of the high temperature, we used an internally cooled electrode. The cerebrospinal fluid space itself plays a protective role against neurotoxicity [9]. Normal saline (0.9%) infusion has been shown to be effective in enlarging the area of necrosis during ablation, acting as a liquid electrode with greater conductivity than that of blood and soft tissues. Furthermore, electrical conductivity is increased even more using a highly concentrated NaCl solution (6-36%) [5,35].

The limitations of this case series study is the small number of patients, short follow-up and, as a new technical purpose, the absence of clear indications. Spinal secondaries in Myeloma Multipla and the presence of anterior location without extended soft tissue involvement may be a possible indication. Neurological involvement and the presence of a tumoral lesion approximately 1 cm from the spinal cord would be a contraindication to this technique.

## Conclusion

The combined image-guided RFA with BKP is a safe, technically feasible, surgical technique, well tolerated by patients, for the treatment of the pathological fractures of the spine. Future prospective studies with larger series and longer follow-up are needed for evaluation of the safety and cost-efficacy of this combined technique.

## Competing interests

The authors have not been influenced by any financial or personal relationship with people or organizations in preparation of this study.

## Authors' contributions

All authors have made substantial contributions to in the design of the article.

PK has contributed to conception and design of the study as well as the final revision and approval of the version to be submitted. PK is the principal surgeon of all the cases. DP has contributed to conception, design, interpretation of data and discussion as well as in surgical technique. KA has contributed in interpretation of data and in surgical technique. AB has contributed in interpretation of data, surgical technique and to follow-up of the patients. GT has contributed to interpretation of histopathological specimens. AK has contributed to revision of the manuscript and to final approval of the version to be submitted.

## Consent statement

Written informed consent was obtained from the patients for publication of this case series and accompanying images. A copy of the written consent is available for review by the Editor-in-Chief of this journal.
